# Perception of medical education by learners and teachers during the COVID-19 pandemic: a cross-sectional survey of online teaching

**DOI:** 10.1080/10872981.2021.1919042

**Published:** 2021-04-19

**Authors:** Emmanuelle Motte-Signoret, Antoine Labbé, Grégoire Benoist, Agnès Linglart, Vincent Gajdos, Alexandre Lapillonne

**Affiliations:** a Université Paris-Saclay, UVSQ, INRAE, BREED, 78350, Jouy-en-Josas, France; b Université Paris-Saclay, UVSQ, UFR Simone Veil Santé, 78180, Montigny le Bretonneux, France; c Neonatal Intensive Care Unit, CHI Poissy St Germain, 78300, Poissy, France; d Ophtalmology, CHU A. Paré, 92100, Boulogne-Billancourt, France; e Department of Pediatrics, CHU A. Paré, 92100, Boulogne-Billancourt, France; f Université Paris-Saclay, Faculté de Médecine, 94270, Le Kremlin-Bicêtre, France; g Department of Pediatric Endocrinology, CHU Bicêtre, 94270, Le Kremlin-Bicêtre, France; h Université de Paris, UFR Médecine Santé, 75006, Paris, France; i Neonatal Intensive Care Unit, CHU Necker Enfants Malades, 75015, Paris

**Keywords:** Online teaching, medical education, health crisis, students’ opinion, teachers’ opinion

## Abstract

COVID-19 lockdowns have deeply impacted teaching programs. Online teaching has suddenly become the main form of medical education, a form that may be used as long as the pandemic continues. We aimed at analyzing how online teaching was perceived by both teachers and learners to help determine how to adapt curricula in the next few years. An anonymous cross-sectional survey of medical students, pediatric residents, neonatal fellows, and their respective teachers was conducted between June and August 2020 to assess feelings about quality, attendance, equivalence, and sustainability of online teaching programs. 146 Students and 26 teachers completed the survey. 89% of students agreed that the offered online teaching was an appropriate way of teaching during the pandemic. Less than half of learners and teachers felt they have received or provided a training of an equivalent level and quality as in usual courses. About one-third thought that this online teaching should continue after the crisis ends. Medical school students had significantly more mixed opinions on online teaching than residents and fellows did. Attendance of learners significantly improved with synchronous online classes (p < 0.001), and among more advanced learners (p < 0.002). Our study is the first of this kind to assess simultaneously the feelings of learners at different levels (medical students, residents, and fellows) and their respective teachers of pediatric on programs taught online. It showed that online programs were perceived as appropriate ways of teaching during the COVID pandemic. Further studies are, however, needed to assess the efficacy of such teaching methods on medical skills and communication capabilities.

## Introduction

The global pandemic due to SARS-CoV-2 has caused unprecedented changes to society, with social-distancing orders taking effect across France beginning in March 2020 [1]. Universities across the world have quickly reacted, announcing immediate closures. Medical education has suddenly been disrupted. Medical teachers have promptly adapted their classes and most of them have offered online classes and tutorials to allow their students to complete their academic year. Maintaining standards in medical education, and minimizing the assessment disruption have been the initial aims of these urgent and sudden changes of ways of teaching [2].

The COVID-19 crisis has highlighted the necessity to develop online learning and virtual education. While the pandemic continues to affect nearly all aspects of our daily lives and seems to be there to last, medical schools will have to continue with this ‘new normal’ way of education [3], trying to satisfy the five elements of the Sloan quality framework for effective online teaching: student satisfaction, learning effectiveness, faculty satisfaction, student access, and institutional cost-effectiveness [4]. Before the crisis, it has been suggested in a systematic review that online learning is equivalent to traditional teaching in terms of knowledge and skills gained, and student satisfaction [5] but most of studies appears to view video and live lectures as competing approaches to face-to-face teaching when, in fact, their relationship could be symbiotic.

Medical education environment had to change rapidly with the COVID-19 pandemic. This worldwide pandemic did not only create the need but also may have provided a chance to accelerate digital transformation of medical education. We have to anticipate further incorporation of online teaching methods in medical schools. Research is, however, needed to maximize its benefits and to have a greater understanding of the perceived advantages and drawbacks by both the learners and teachers.

During the pandemic, manuscripts have described the kinds of medical curriculum layouts, both for teaching and assessment, from teachers’ and learners’ point of view [6–9]. One publication assessed simultaneously both teachers’ and students’ perspectives [10] but to our knowledge, no studies have reported surveys assessing teachers’ and students’ perspectives according to the degrees of education. We, therefore, designed a cross-sectional study of the perception of both learners and teachers on online teaching evaluating global satisfaction, attendance, feeling of equivalence, and overall pros and cons, taking into account the degree of education.

## Materials and methods

### Participants and course description

We designed a study to survey three different groups of learners registered in three medical school of the Paris region and their respective teachers all teaching pediatrics. In France, pediatrics is taught to all medical students during the 4^th^ or 5^th^ year of medical school. Pediatrics is also taught during residency to students who want to graduate in pediatrics. Finally, subspecialties in pediatrics are taught during the fellowship program to young pediatricians who want to become neonatologists for example.

Coordinators of the pediatric programs are elected for a five-year term. In the Paris region, there are six medical schools, one pediatric residency, and one neonatal fellowship program. As a consequence, and in order to cover all pediatric courses delivered to medical students, residents and fellows, coordinators of each program, who belong to different three different medical schools, were informed of the study. They were asked for permission to contact both the students and teachers. The study received approval from the three Universities’ student councils.

### Type of courses before and during the lockdown

Courses before the lockdown were classically face-to-face lectures and tutorials, with inconstant access to slide-based presentations and seldom use of online teaching. Besides theoretical courses which took place mainly during the afternoons, practical and clinical teaching was provided as internship, residency, or fellowship, respectively, within the hospital wards mainly during the mornings and/or on duty shifts. During the lockdown period, three types of online courses have been proposed to learners: slide decks, prerecorded slide-based lectures, and webinars using various platforms (synchronous live online classes) allowing interactions with the teachers (voice or chat), real-time video and data transfer between two or more groups [11]. For the purpose of the study, the slide-decks and the prerecorded slide-based lectures were regrouped in one type of teaching called asynchronous presentations, whereas webinars were called synchronous live classes.

The three types of courses were provided to fellows, webinars, and prerecorded slide-based lectures were offered to residents and slide-deck presentations and prerecorded slide-based lectures were offered to medical students. Practical and clinical teaching did not changed during this period and was similar to that before the lockdown.

### Methods

All current registered 5^th^ year medical students of one medical school of the Paris region, and all pediatric residents and neonatal fellows of the Paris region were sent an online link to a survey to be filled online. Similarly, teachers were asked to respond to another online specifically designed for them. Participation to the survey was voluntary, and participants were informed prior to starting the survey that all data were collected anonymously. Surveys were opened from 15 June 2020 to 15 August 2020. The link was resent once before closure of the study. The survey was designed by two investigators and carried eight questions for the learners and eight for the teachers (the detailed surveys are provided in supplementary data). It was designed to be completed in 5 min or less.

The goal of the survey was to provide a cross-sectional view into the perception of online education from both teachers and students. Since validated survey did not exist in this field, we used the 5-points Likert scale as a standard procedure [12]. The items assessed the type of online teaching, the attendance to the program, the perception of the appropriateness of the course with regard to the health crisis, how it compared to the usual course, and whether or not it could be sustained after resolution of the crisis. Answering this first series of closed questions was mandatory. The survey also included open questions allowing participants to point out the pros and cons of each type of online courses but these questions were not mandatory. All answers were considered in the data analysis.

### Statistical analysis

Data were managed using Microsoft Excel. Cross-tabulated frequencies and percentages were calculated, and all associations were quantified using chi-squared and Fisher’s exact tests, as applicable. Participants identified their level of agreement or disagreement on a five-point Likert scale, and a proportional odds model was used to analyze the expressed statements. A p-value equal to or less than 0.05 was considered to indicate a statistically significant relationship between two variables.

## Results

### Population of respondents

A total of 172 responses were collected, 146 from learners and 26 from teachers. There was no significant difference in response rate between learners and teachers, or within groups of learners (Table 1).

**Table 1.** Response rate according to type of respondents

	Responses	Response rate	p
Teachers n = 69	26	37.7%	ns
Learners n = 326	146	44.8%	
Medical students (n = 131)	54	41.2%	ns
Residents (n = 82)	23	28.0%	
Fellows (n = 113)	68	60.2%	

ns = non significant

### Type of online teaching and attendance of learners

Among the 146 responding learners, 71.9% were offered pre-recorded lectures with no live interaction and 91 62.3% were offered live online classes with possibility of live chat and/or communication with the teacher. Attendance of learners was overall poor with 22% to 54% of the learners having not followed or followed less than half of the online courses. Attendance was significantly greater for live classes than for asynchronous presentations (p < 0.001) and was greater for fellows, residents, and medical students (p < 0.002 and p < 0.001 respectively) (Table 2).

**Table 2.** Attendance of learners according the type of online teaching

	Asynchronous presentations (n = 105)			Synchronous live classes(n = 91)			
	Students (n = 54)	Fellows (n = 51)	p <0.001	Residents (n = 23)	Fellows (n = 68)	p<0.002	p <0.001
None	14 (26%)	0 (0%)		0 (0%)	1 (1%)		
Less than half	15 (28%)	20 (39%)		5 (22%)	10 (15%)		
More than half	17 (31%)	19 (37%)		10 (43%)	16 (24%)		
All	8 (15%)	12 (22%)		8 (35%)	41 (60%)		

For the purpose of the study, the slide-decks and the prerecorded slide-based lectures were regrouped in one type of teaching called asynchronous presentations, whereas webinars were called synchronous live classes

### Perception of the appropriateness of the course with regards to the health crisis

Eighty-nine percent of students strongly agreed or agreed that online teaching was an appropriate way of delivering courses during the COVID-19 pandemic with no significant difference of responses among medical students, residents, and fellows (85.5%, 95.7%, and 89.7%, respectively).

### How do online courses compare to the usual courses?

**Figure 1.**
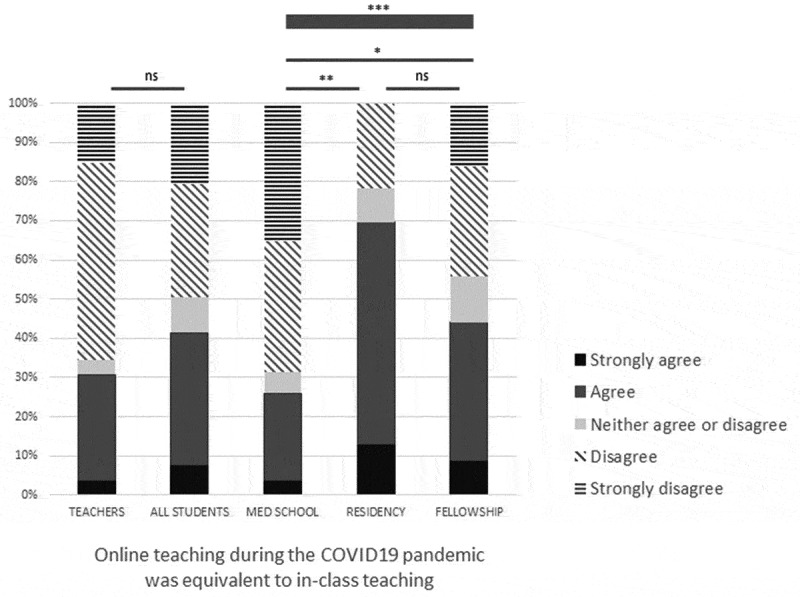
Opinions about equivalence of online vs in-class courses according to type of respondents

Figure 1 illustrates the responses regarding the feeling of equivalence between online and in-classes, and the desire to continue with online courses. The majority of learners (58.6%) and teachers (69.2%) feel they have not received or provided a theoretical training of an equivalent level and quality as expected. There is no significant difference between students’ and teachers’ feelings. Among learners, the medical students were those who disagreed or strongly disagreed the most that online and in-class teaching were equivalent (Figure 1).

Thin line = Fisher’s test between two groups; thick line = Chi^2^ test between three groups. ns = non-significant, *** = p < 0.001, ** = p < 0.01, * = p < 0.05

### Should online teaching continue after resolution of the crisis?

**Figure 2.**
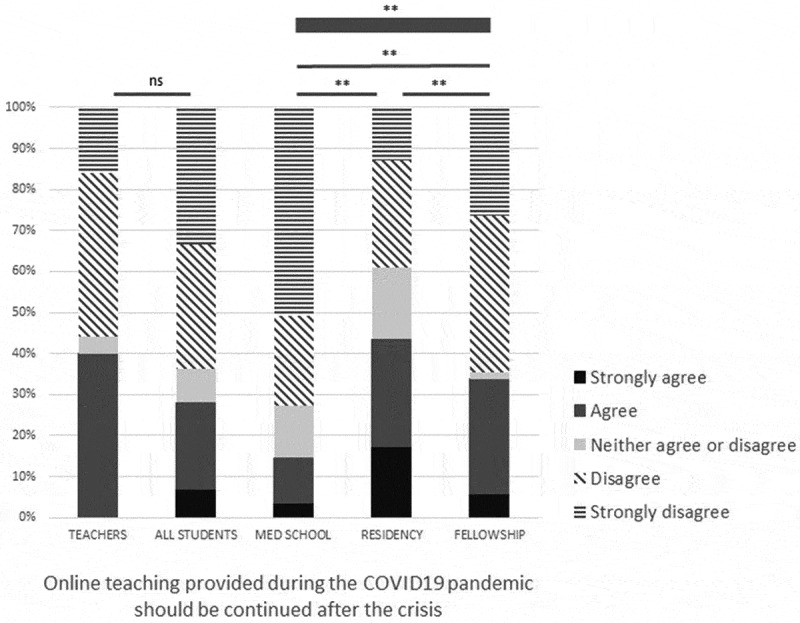
Opinions about sustainability of online courses after resolution of the health crisis according to type of respondents

About one-third of the respondents (28.0% of learners and 38.5% of teachers) thought that this kind of online curriculum should continue after resolution of the crisis. There were significant differences of feeling among types of learners, medical students disagreeing or strongly disagreeing with the proposal of continuing online teaching after the crisis has resolved (Figure 2).

Thin line = Fisher’s test between two groups; thick line = Chi^2^ test between three groups. ns = non-significant, ** = p < 0.01

### Pros and cons of each type of online courses

Among the 51 learners who answered the open question ‘to your opinion, were prerecorded lectures of sufficient quality for theoretical learning?’, 78% agreed or strongly agreed. Overall the learners expressed congratulations of the teachers’ reactivity for having rapidly adapted the curriculum to the difficulties encountered because of the health crisis. For the pros, residents and fellows mentioned a gain of time, and all learners liked having the option to play back the lectures. On the other hand, the cons expressed by the learners were concentration issues, absence of interaction with the teachers and their peers, and repeated technical difficulties (Wi-Fi issues for example).

Of the 26 teachers who have responded to the open question, 26 (76.9%) declared that synchronous online classes are a more appropriate way of teaching. They expressed repeatedly that they prefer live online classes to pre-recorded lectures and that online teaching is more adapted to residents and fellows than medical students who benefit the most of the interactions with their lecturer. Among pros expressed by the teachers were improved quality of lessons because of the need to specifically adapt the course to online teaching, gain of time (no transit time between hospital and university for example) and possibility for students to playback the lecture. Poor interaction (particularly with the repeated term ‘dehumanization’) and absence of or poor feedback from learners were the cons raised by the teachers.

## Discussion

The purpose of this study was to assess the perception of online teaching by learners and teachers of medical education that has dramatically changed because of the lockdown of the universities during the COVID-19 pandemic. Our study aimed to deeply assess student and faculty satisfaction, two of the five pillars of the Sloan Consortium (student satisfaction, learning effectiveness, faculty satisfaction, student access, and institutional cost-effectiveness) [13]. A large majority of learners agreed that online teaching was an appropriate way of teaching during the health crisis and largely congratulated the educational staff for its reactivity. Nevertheless, less than half of learners felt they have received a training of an equivalent level and quality as in usual courses and only one-third thought that this kind of curriculum should continue after the crisis.

In a previous study, online learning before the COVID-19 pandemic seems to be equivalent to traditional teaching in terms of knowledge and skills gained, and student satisfaction [5] and it appears that students prefer synchronous live lectures. Since the beginning of the COVID-19 pandemic, some manuscripts reported points of view of the situation from either learners [6,8,9,14,15] or teachers [2,7,11,16,17]. Outcomes in students’ satisfaction were variable but distinction between the different forms of online teaching was not made [8,9]. Nevertheless, they consistently seem to prefer synchronous live lectures [18], as it was already reported before the crisis [19–21]. A single paper presents an experience of moving the classes online and the survey of students and professors for their feedback. Students were generally satisfied with the online course. As also shown by our study, learners preferred online to offline lectures but contrary to our study, they wanted to maintain the online course after the end of the pandemic [10].

It is obvious that within the emergency of implementation of online teaching for most of the universities, some of the provided courses have potential for improvement. Concerns have been raised regarding the quality of resources produced during the pandemic because of time constraints. For example, faculty will have to deal with lack of motivation and difficulty concentrating for some students who prefer face-to-face teaching. Teachers’ feelings are pretty much the same as the students, and they highlight that the type of online teaching should be adapted to the target audience, always encouraging synchronous online classes to maintain interactions between learners and teachers. Moreover, sudden shift toward the use of online teaching on a large scale have led to inconsistencies with medical curricula, many teachers being inadequately prepared [8]. Considering the fact that remote learning is going to become the major way of theoretical education for at least several months, there will be an urgent need for teachers training [22–24].

Our study also highlighted key perceived advantages of online education, reinforcing those previously described. For the learners, it allows a gain in time, costs, and flexibility because they can learn at their own pace [8]. The most popular use of asynchronous presentations is for revision or recap of difficult topics, students being able to access teaching material when they want to [21]. Teachers have discovered the efficiency, ease of access, and diverse collaborations afforded by the virtual world, and many aspects of online teaching will likely continue even after relaxation of pandemic-related societal restrictions [25].

Our results also showed poor attendance of the learners, especially medical students and when prerecorded lectures were provided. Teaching staff organized these adaptations to courses in a hurry and did not anticipate a need to check attendance or continuous assessment of knowledge. There is no doubt it can be improved, especially for medical students who may need a closer educational support than residents and fellows. Differences about acceptation of online teaching in function of students’ maturity has been previously reported and showed that younger students may benefit more from live lectures due to inexperience with managing their workload by themselves. On the other hand, and as suggested by our study, more advanced students may prefer more freedom to manage their workload between hospital and lectures [21]. It would be interesting to develop peer training groups [26] in order to limit learning dropout, and improve the accessibility to lessons.

We acknowledge that our study has several limitations. Overall, the response rate was less than 50% and we cannot exclude a response bias. We may have not surveyed some groups of students, particularly those who have no free and/or easy access to internet, and/or those who are not motivated by online interactions. However, since we have surveyed a large number of learners and teachers, we believe that the data that we have obtained bring to light some significant conclusions. Our study assessed the perception of online teaching for theoretical medical knowledge but universities will also have to deal with skills and communication tools for the medical training of their students, which have been, due to COVID-19, partly excluded of the direct relationship with the patient [7,27,28]. Since the practical and clinical teaching was similar before and during the lockdown, we hypothetize that this aspect of the medical training has not been affected by the pandemic, recognizing, however, that we have not assessed it specifically. By study design, we also did not assess either the objective quality of the provided teaching or the efficiency of the training. Finally, we have not studied the feasibility and perception on online examination from home. This may remain a possibility if the pandemic persists since it has been shown that open-book examination seems reasonably satisfactory in assessing the knowledge of students [29,30]. We should also consider the fact that our survey has been provided just after a crisis situation and that the answers to the survey could be difficult to extrapolate to a usual curriculum.

As the months pass, we have no other choice but to realize that medical education is going to be a long-term challenge and that the health situation will not permit a rapid ‘back-to-normal’ way of life. Whereas emergency decisions had to be taken in March 2020 after closure of universities and disruption of usual curriculum to allow the students to complete the academic year remotely, there is an urgent need to optimize online teaching, which was already being implemented in Medical Schools all over the world during the last decade for every degree of medical education [17,31,32]. The pandemic does not only create the need but rather may provide the chance to accelerate digital transformation in medical education. The term ‘new normal’ has been repeatedly used with some advices for the faculty to adapt their courses [3,22,25,33], online curricula having a predominantly positive perspective if combined with face-to-face learning [34]. These changes may also provide a unique opportunity for interaction and partnerships between universities, which can be particularly useful for those who do not have the capabilities to rapidly implement such transformation [35–37].

## Supplementary Material

Supplemental Material
