# Supplementary material for: Perception of medical education by learners and teachers during the COVID-19 pandemic: a cross-sectional survey of online teaching
Source: Med Educ Online. 2021 Apr 19;26(1):1919042. doi: 10.1080/10872981.2021.1919042 (PMC8079026; doi:10.1080/10872981.2021.1919042)
Supplement: Supplemental Material [file ZMEO_A_1919042_SM1139.docx]

**SUPPLEMENTARY DATAS**

**SD1: learners’ survey**

Your teaching staff has offered an online curriculum containing prerecorded lectures and live online classes for your pediatrics training. Did that choice appear adapted to the health crisis?

Strongly agree

Agree

Neither agree nor disagree

Disagree

Strongly disagree

To your opinion, were prerecorded lectures of sufficient quality for theoretical learning?

Strongly agree

Agree

Neither agree nor disagree

Disagree

Strongly disagree

How many of the provided prerecorded lectures did you attend?

All

More than a half

Less than a half

None

How many of the provided live online classes did you attend?

All

More than a half

Less than a half

None

If you answered « all » or « more than a half » to the two previous questions, what did you especially like? Open-ended answers

If you answered « none » or « less than a half » to the two previous questions, what was (were) the reason(s)? Open-ended answers

To your opinion, was online teaching during COVID-19 pandemic equivalent to in-class teaching?

Strongly agree

Agree

Neither agree nor disagree

Disagree

Strongly disagree

To your opinion, should online teaching during COVID-19 pandemic continue after the health crisis?

Strongly agree

Agree

Neither agree nor disagree

Disagree

Strongly disagree

**SD2: teachers’ survey**

During COVID-19 lockdown, which target audience(s) did you teach?

Pre-Med School

Med School

Residents

Fellows

Other curriculum

In what form(s) did your teaching program take place?

Online lecture without sound

Recorded online lectures

Live online classes

Cancelation

Other (open-ended answer)

A posteriori, which form of teaching is the more adapted?

Online lecture without sound

Recorded online lectures

Live online classes

Cancelation

Other (open-ended answer)

To your opinion, was online teaching during COVID-19 pandemic equivalent to in-class teaching?

Strongly agree

Agree

Neither agree nor disagree

Disagree

Strongly disagree

To your opinion, should online teaching during COVID-19 pandemic continue after the health crisis?

Strongly agree

Agree

Neither agree nor disagree

Disagree

Strongly disagree

Would your answers to the two precedent questions be the same for every kind of target audience?

Yes

No

If you answered yes, for which kind of students is online teaching more adapted?

Pre-Med School

Med School

Residents

Fellows

Other curriculum

Give 2-3 pros and cons for your experience of online teaching (Open-ended answers)
